# Validity and Reliability of an Immersive Virtual Reality System for Multidimensional Assessment of Cervical Sensorimotor Control: Cross-Sectional Study

**DOI:** 10.2196/88498

**Published:** 2026-05-21

**Authors:** Ya-Lan Chiu, Pei-Yun Lee, Pooi-Ling Lim, Kai-Chia Cheng, Zong-Xian Yin, Yi-Ju Tsai

**Affiliations:** 1 Department of Physical Therapy College of Medicine National Cheng Kung University Tainan Taiwan; 2 Physical Therapy Center National Cheng Kung University Hospital Tainan Taiwan; 3 Institute of Allied Health Sciences College of Medicine National Cheng Kung University Tainan Taiwan; 4 National Center for Geriatrics and Welfare Research National Health Research Institute Yunlin Taiwan; 5 Department of Computer Science and Information Engineering Southern Taiwan University of Science and Technology Tainan Taiwan

**Keywords:** cervical spine, neck pain, proprioception, reliability, sensorimotor control, validity, virtual reality

## Abstract

**Background:**

Cervical sensorimotor control (SMC) is often disrupted in individuals with chronic neck pain, contributing to persistent symptoms and functional limitations. Traditional cervical SMC assessments are limited by complex setups, single-domain testing, and examiner dependency. Virtual reality (VR) technology offers a promising platform for multidimensional, standardized, and user-friendly assessment.

**Objective:**

This study aimed to develop and evaluate the validity and reliability of a VR-based system for assessing cervical SMC in healthy adults.

**Methods:**

A cross-sectional observational study was conducted in 30 healthy adults (aged 18-60 years). The custom-developed VR system (HP Reverb G2 Omnicept Edition, Unity engine) incorporated 5 SMC tests: cervical range of motion (ROM), joint position error (JPE), head-tilt response, figure of eight (FOE), and postural sway (PS). Test-retest reliability was assessed across 2 sessions, separated by 1 week, using intraclass correlation coefficients (ICCs). Concurrent validity was examined by comparing VR-based measures with gold standard optical motion capture or established clinical tools using Pearson correlation coefficients.

**Results:**

The VR system demonstrated good to excellent test-retest reliability across most outcome measures. The ICC for cervical ROM ranged from 0.851 to 0.968 across movement directions. The ICC for JPE in each direction ranged from 0.813 to 0.827. The ICC for the FOE test’s deviation frequency and task duration were 0.810 and 0.913, respectively. The ICC for the head-tilt response was 0.742 and ranged from 0.720 to 0.843 for PS under both visual conditions. The VR-based assessments for ROM, JPE, FOE, and PS showed strong correlations with reference measures (*r*=0.723-0.980), supporting concurrent validity.

**Conclusions:**

This VR-based assessment system provides a valid, reliable, and user-friendly multidimensional evaluation of cervical SMC. It offers a standardized, integrated, and clinically feasible alternative to conventional assessments, with potential applications in both clinical diagnostics and rehabilitation monitoring.

**Trial Registration:**

ClinicalTrials.gov NCT06474130; https://clinicaltrials.gov/study/NCT06474130

## Introduction

Neck pain is a common global health concern, with epidemiological studies suggesting that approximately 30% to 50% of adults experience it at least once in their lifetime [1,2]. Although many acute episodes resolve spontaneously, a considerable proportion of individuals develop chronic neck pain (CNP), typically defined as pain persisting for more than 3 months [3]. Recent estimates suggest that more than 200 million people worldwide are affected by CNP, making it one of the leading causes of musculoskeletal disability globally. The prevalence of CNP is expected to rise sharply in the coming decades due to population aging and sedentary lifestyles [2,4]. Beyond physical discomfort, CNP is associated with psychological distress, decreased work productivity, and substantial long-term health care expenditures [5,6]. Moreover, evidence suggests that individuals with CNP frequently exhibit various sensorimotor control (SMC) impairments, including impaired proprioception, delayed neuromuscular responses, reduced joint mobility, altered eye-head coordination, and increased movement variability and postural sway (PS), which may complicate rehabilitation efforts and contribute to the persistence of symptoms [7-11].

Accurate assessment of cervical SMC is important for identifying underlying deficits associated with CNP and tailoring rehabilitation strategies. Previously, assessment methods targeting distinct functional components of cervical SMC were established and used in both clinical and research settings [7,12,13]. However, they often require multiple instruments, experienced examiners, and controlled environments. For example, cervical range of motion (ROM) assessments vary in complexity, from the use of basic tools such as goniometers and inclinometers to more advanced systems including optical motion capture and inertial measurement units (IMUs) [14]. The joint position error (JPE) test for cervical proprioception typically requires a head-mounted laser pointer and a target board to quantify repositioning error [9,10,15]. PS, which reflects whole-body balance control, is usually measured using force platforms or stabilometric systems [16]. Their fragmented nature and susceptibility to human error limit their clinical scalability. Therefore, there is an increasing need for more accessible, integrated, and standardized approaches to comprehensively assess cervical SMC across its multiple dimensions.

Virtual reality (VR) technology enables immersive, interactive, and quantifiable testing environments that integrate multiple SMC domains [17-20]. Head-mounted VR systems with embedded motion sensors enable real-time tracking of head kinematics, providing standardized and automated feedback [18,21]. Recent research has increasingly explored the potential of VR as a tool for assessing cervical SMC in individuals with CNP, although the studies are still limited. Few studies have validated VR-based measures of cervical motion and proprioception, showing strong agreement with gold-standard systems [10,22-24]. The VR-based assessment of the subjective visual vertical or head-tilt response (HTR) has also been shown to be reliable for screening otolithic dysfunctions and measuring perceptual orientation [25-27]. These findings suggest that VR may offer a compelling, multidimensional platform for the precise and engaging assessment of SMC in both clinical and research settings. However, current studies still focus on a single domain of SMC and lack integrated evaluation across multiple sensorimotor components. To address this gap, this study developed a multidimensional immersive VR-based cervical SMC assessment system that integrates mobility, proprioception, coordination, verticality perception, and postural stability within a single protocol. The study aimed to (1) evaluate the test-retest reliability of the VR system and (2) examine its concurrent validity compared with established reference methods. We hypothesized that the VR-based assessments would demonstrate good to excellent reliability (intraclass correlation coefficient [ICC] ≥0.75) and strong correlations (*r*≥0.70) with gold-standard measures.

## Methods

### Study Design

This cross-sectional observational study was conducted from October 2024 to May 2025 in a university laboratory.

### Participants

A total of 30 healthy adults (15 male and 15 female; mean age 20.43, SD 1.01 years; mean height 166.08, SD 8.57 cm; mean weight 59.38, SD 12.49 kg) participated in the study. Exclusion criteria included (1) current spinal pain and spinal deformities, (2) history of spinal fracture or surgery, (3) neurological or musculoskeletal conditions that may affect performance, (4) diseases causing balance dysfunction such as central or peripheral nervous system disorders, or vestibular system dysfunction, (5) metabolic diseases, (6) psychological conditions that may affect questionnaire responses, and (7) visual impairments requiring correction, if participants were unable to wear contact lenses during VR testing.

Power analysis (α=.05; power=0.90) indicated that a minimum of 24 samples was required for reliability (expected ICC=0.75) and 14 for validity (expected *r*=0.70), ensuring adequate power for all analyses [28,29]. The sample size was estimated using G*power software (version 3.1.9.6; Heinrich Heine University Düsseldorf).

### Instrumentation

#### VR-Based System

**Figure 1:**
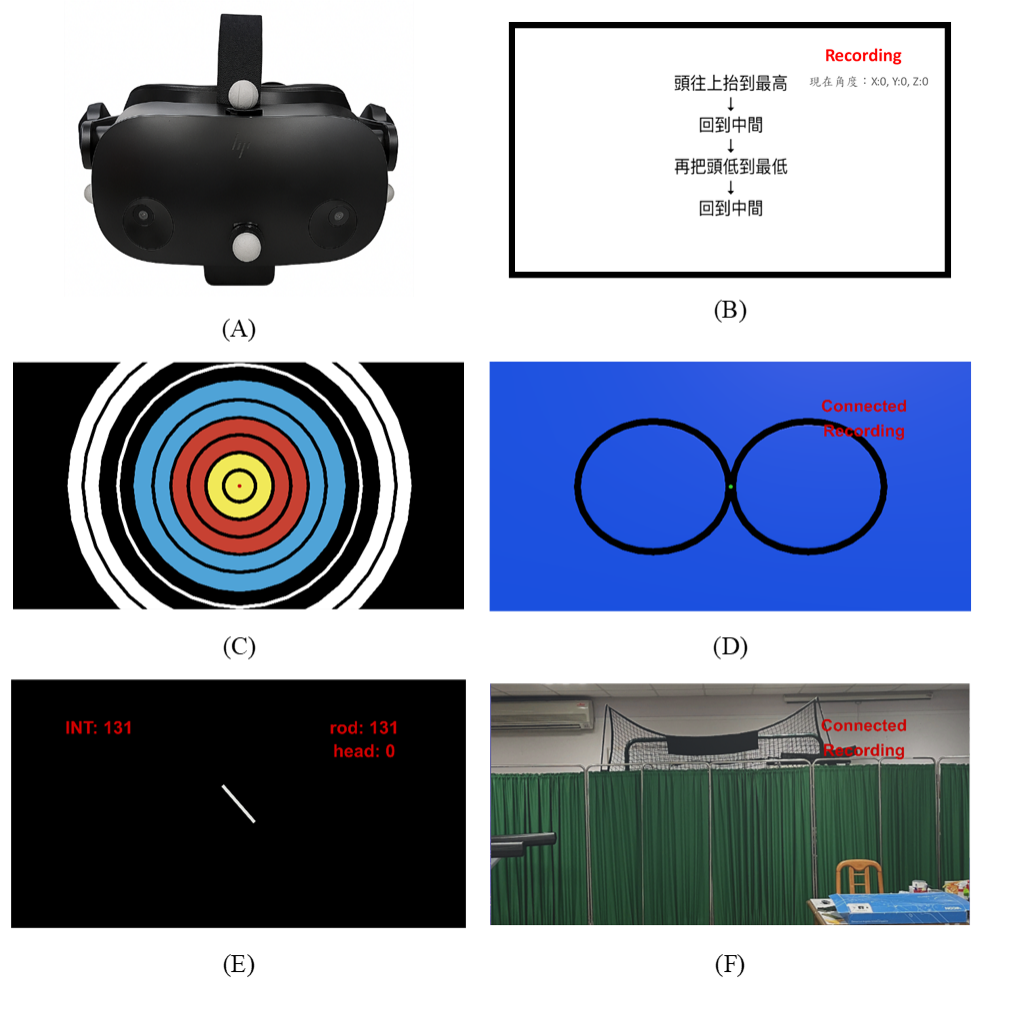
Virtual reality system used for cervical sensorimotor control assessment. (A) HP Reverb G2 Omnicept Edition head-mounted display and the reflective markers attached for the validity study. (B) Virtual environment designed for cervical range of motion assessment. (C) Virtual environment designed for joint position error assessment. (D) Virtual environment designed for figure-of-eight assessment. (E) Virtual environment designed for head-tilt response. (F) Virtual environment designed for postural sway assessment.

A custom VR-based cervical SMC assessment system was developed using the HP Reverb G2 Omnicept Edition headset (HP Inc; Figure 1A), integrated with a custom-built virtual environment created using Unity 3D (Unity Technologies). The VR headset is equipped with embedded IMUs (gyroscope, accelerometer, and magnetometer) operating at a sampling rate of 512 Hz, capturing 3D inertial signals to enable precise tracking of head kinematics. The system incorporated 5 virtual test modules simulating standardized SMC assessments: (1) ROM tests to determine maximal active motion range, (2) JPE test to quantify cervical proprioceptive accuracy, (3) figure of eight (FOE) test to assess visual-head dynamic coordination, (4) HTR to assess verticality perception, and (5) PS to assess postural stability in standing postures. All modules provided real-time visual feedback and standardized instructions. All visual targets and feedback across modules were presented as virtual images within the VR environment at a fixed virtual distance. No real-world targets or pass-through vision were used.

#### Reference Systems

To evaluate concurrent validity, the VR-derived outcomes were compared with those obtained from a motion analysis system or a clinical observation test. An optical motion capture system, including 10 cameras (VICON T10, Vicon Motion Systems Ltd) with a 100 Hz sampling rate, was used as the gold standard for ROM, JPE, and PS measures. A total of 4 reflective markers with 14 mm spheres were attached onto the front, top, left, and right sides of the VR headset to define a local coordinate system of the head (Figure 1A). Trajectory data were first digitized using the Nexus 2.10 software (Vicon Motion Systems Ltd) and subsequently processed using a custom MATLAB script (R2023a, MathWorks Inc).

### Experimental Procedures

**Figure 2:**
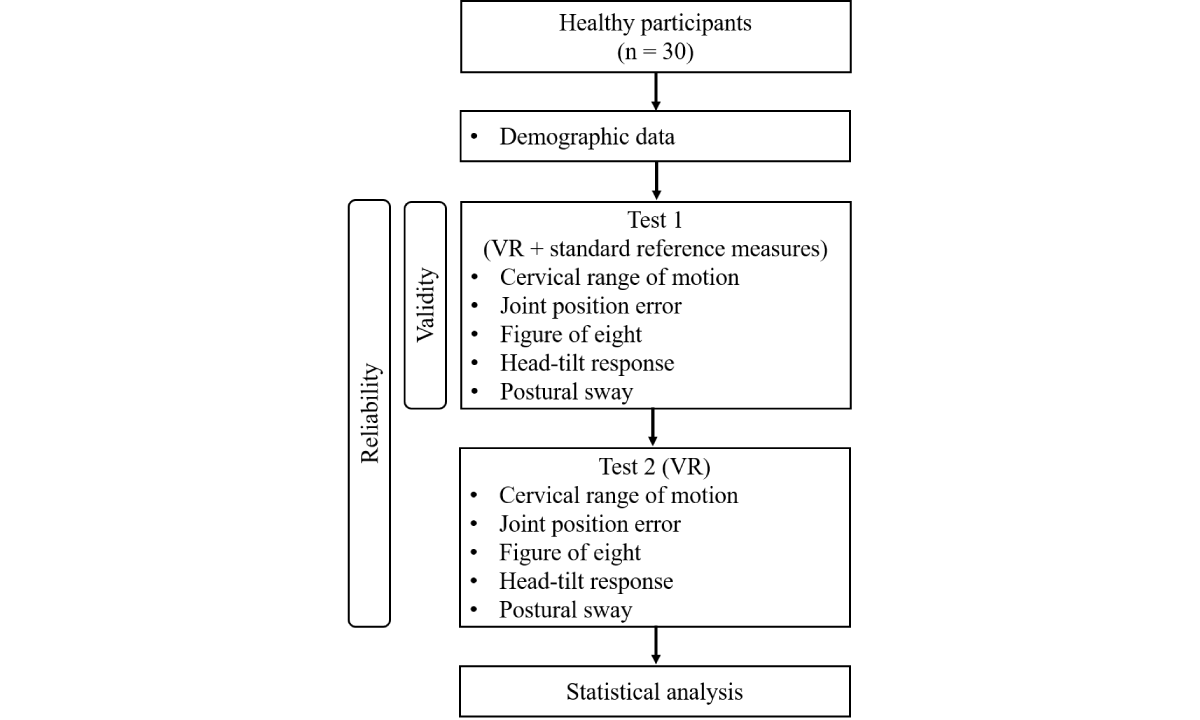
Study design and overall experimental procedures. VR: virtual reality.

Participants completed 2 identical VR assessment sessions, separated by 1 week, to assess test-retest reliability. To examine validity, VR and reference measures were collected concurrently within the same first session (Figure 2). All participants wore the VR headset in a comfortable and stable position throughout the assessment. For most tests, they were seated in a back-supported chair with their feet flat on the ground and their hips/knees at approximately 90 degrees. The only exception was the PS assessment, which was performed in a quiet standing position with feet together. During all seated tasks, participants were instructed to maintain their trunk in contact with the back support and to avoid any trunk or shoulder movement during all head motion tasks. The examiner visually monitored participant performance throughout testing, and trials were repeated if obvious compensatory trunk or upper body movements were observed.

To ensure consistency in orientation-based measurements, a neutral head position was established through a standardized calibration procedure. Participants were instructed to adopt a comfortable, upright head posture while looking straight ahead at a fixed visual reference in the virtual environment. Head orientation data were recorded over a brief 1-second static period, and the average orientation during this period was defined as the neutral reference. This calibration procedure was repeated before each trial to minimize the influence of sensor drift or initial positioning offsets. The same standardized instructions and procedures were applied across sessions to ensure consistency between test and retest measurements.

Participants completed a standardized familiarization phase prior to formal data collection. Each participant performed one practice trial for each task. Only for the HTR task, 2 practice trials were provided due to its greater complexity and to ensure adequate understanding. No performance feedback was provided beyond standardized instructions. A 1-minute rest period was provided between tests to minimize fatigue. The same testing procedures and environmental conditions were maintained across sessions to ensure consistency. All assessments were administered by a single trained examiner who provided standardized instructions using a predefined script. The same examiner conducted both test sessions for each participant to ensure consistency across sessions. Given the automated nature of the VR system, examiner involvement was limited to task instruction and safety supervision, with no manual data recording or subjective scoring involved. The examiner was blinded to the results of the previous session.

### Five Cervical SMC Assessments

#### ROM Assessment

Cervical ROM tests were assessed to evaluate movement capacity in 6 directions: flexion, extension, lateral bending, and rotation to both the left and right sides [30]. Participants followed visual cues in the VR headset to move their head to the maximal range in each direction and then return to neutral (Figure 1B). Each movement was performed 3 times, with rest between directions to prevent fatigue.

For VR data analysis, head orientation was continuously tracked using the headset tracking system and expressed as an Euler angle sequence relative to the calibrated neutral position. Orientation data were derived from quaternion-based signals integrated from inertial and positional tracking and converted within Unity (transform.eulerAngles). Data were then exported and processed offline using MATLAB (MathWorks), where angular discontinuities were corrected, and values were expressed relative to the initial position. No additional filtering was applied. Peak angular displacement was identified for each trial, and the mean of the 3 trials was used as the final ROM value [31,32].

For concurrent validation, cervical ROM was simultaneously captured using the Vicon optical motion system. Euler angles were computed from the head orientation relative to the global coordinate system, and peak angular displacements in each direction were identified using the same criteria as those applied to the VR-based data.

#### JPE Assessment

The JPE test evaluated cervicocephalic proprioception by measuring head repositioning accuracy in the absence of visual feedback [33,34]. In the VR headset, a virtual target (50 cm in diameter with concentric rings at 5 cm intervals) was displayed 1 meter in front of the participant (Figure 1C). Participants were instructed to align a red dot representing the head orientation with the target center, memorize the starting position, close their eyes, rotate their head in 1 of 4 directions (flexion, extension, right rotation, or left rotation), and attempt to return to the starting point. Three trials were completed per direction, and mean values were used for analysis [12,14].

For VR data analysis, head orientation data were continuously tracked by the embedded IMUs. The orientation data were transformed into a forward unit vector to derive 2-dimensional coordinates of the red dot. No additional filtering was applied. The difference between the starting and repositioned coordinates was converted into an angular deviation, representing repositioning error.

For concurrent validation, head orientation during the JPE test was simultaneously recorded by the Vicon motion capture system. JPE values were calculated as the angular difference between the head’s initial orientation and return orientation, following the same computational criteria used for the VR data.

#### FOE Assessment

The FOE test assessed sensorimotor coordination, as well as the smoothness and accuracy of head-controlled tracking [35,36]. In the VR environment, a horizontal FOE path (2 cm wide) appeared 1 meter in front of the participant, with a red dot pointed to the center of the visual field serving as the controllable cursor (Figure 1D). Participants traced the figure continuously using head movements at a self-selected comfortable speed. Each participant performed 3 trials, and mean values were used for analysis.

For VR data analysis, the system recorded the cursor trajectory in a 2-dimensional X-Y plane. Two primary metrics were derived, including the completion duration and the number of deviations. Completion duration was the total time required to complete one full tracing path. The number of deviations was the total number of instances when the red dot moved outside the designated path for at least 1 consecutive frame. Continuous frames outside the boundary were counted as a single deviation until the red dot reentered the path.

For clinical validation, participants performed an equivalent head-controlled tracing task using a laser pointer mounted on the top of the head to trace a physical FOE pattern (2 cm wide, size 85 × 38 cm) displayed on a vertical board positioned 1 meter in front, replicating the VR environment conditions. Outcome measures, including completion duration and number of deviations, were manually recorded by an assessor using a stopwatch and visual inspection of the recorded videos.

#### HTR Assessment

The HTR test evaluated verticality perception and cervical proprioceptive control, reflecting visual dependency in spatial alignment. In the VR environment, a 15 cm white rod was displayed against a black background and randomly tilted between 45° and 135°, in 1° increments (Figure 1E). Participants were instructed to laterally tilt their heads to align the rod to the perceived vertical, after which the system automatically recorded the final orientation. A total of 20 trials were performed, and the mean angular deviation across trials was calculated as the outcome measure.

For VR data analysis, head orientation was continuously tracked via embedded IMUs. The system calculated the angular deviation between the participant-adjusted rod and the true vertical reference to quantify perceived alignment error. Mean absolute deviation across all trials represented the final HTR score.

The HTR test was excluded from the validity analysis since it primarily measures perceptual-cognitive processing rather than biomechanical movement. It reflects verticality perception and multisensory integration based on internal psychophysical judgments, and has previously demonstrated acceptable reliability and validity in computerized or virtual platforms [25,26].

#### PS Assessment

The PS test assessed whole-body balance control and postural stability under eyes-open and eyes-closed conditions [16,27,37]. Participants stood upright with their feet together and arms at their sides for 20 seconds while viewing a static virtual scene that replicated their natural environment (Figure 1F). Three trials were performed per condition, and mean values were used for analysis.

For VR data analysis, head motion was continuously recorded by the IMUs of the headset, which measured linear acceleration in the anteroposterior and mediolateral directions as an indirect measure of PS [38,39]. Acceleration data were processed using standard signal processing procedures, including unit conversion, low-pass filtering (6 Hz), and smoothing to reduce high-frequency noise. Sway trajectories were reconstructed using signal vector magnitude and direction cosine transformations [39]. Based on an estimated head height [40], linear displacements in the anteroposterior and mediolateral directions were derived. Four sway parameters were obtained to quantify balance performance, including maximum anteroposterior and mediolateral displacements, total path length (TPL), and sway area (SA) [40-42].

For concurrent validation, Vicon motion capture data were used to estimate head center position from the midpoint between reflective markers, serving as a proxy for the body’s center of mass. The X and Y coordinates of this midpoint represented mediolateral and anteroposterior displacements, respectively. The same sway parameters, including maximum anteroposterior and mediolateral displacement, TPL, and SA, were computed to enable cross-system comparison of postural stability performance.

### Statistical Analysis

Test-retest reliability of the VR-based system was examined using the ICC with the 2-way random-effects model (ICC_2,1_). Reliability was interpreted as poor (<0.50), moderate (0.50-0.75), good (0.75-0.90), and excellent (≥0.90) [43]. The standard error of measurement (SEM) was calculated to estimate score precision, and the minimal detectable change (MDC) was derived to identify the smallest change exceeding measurement error. Agreement between sessions was further evaluated using Bland-Altman plots, illustrating the mean difference and 95% limits of agreement (mean ± 1.96 × SD) [44]. Concurrent validity was assessed using Pearson correlation coefficients (*r*) between VR-based outcomes and corresponding measures from clinical tests or the Vicon motion capture system. Correlation strength was interpreted as poor (<0.30), fair (0.30-0.49), moderate (0.50-0.69), strong (0.70-0.89), or excellent (≥0.90) [45]. All statistical analyses were performed using IBM SPSS Statistics 25 (IBM Corp). In line with recommendations for measurement studies, ICC and Pearson correlation coefficients were interpreted as measures of effect size, and emphasis was placed on the magnitude and precision of estimates (eg, 95% CI, SEM, and MDC) rather than statistical significance testing.

### Ethical Considerations

The study was prospectively registered on ClinicalTrials.gov (NCT06474130). Ethics approval has been received from the institutional review board of the National Cheng Kung University Hospital (B-ER-113-126). Written informed consents were obtained from all participants before the experiment. Participant data were handled with strict confidentiality. All identifiable information was removed prior to analysis, and the data were stored securely with access limited to members of the research team. No financial compensation was provided to participants for their involvement in this study.

## Results

### Test-Retest Reliability

The VR-based assessment system demonstrated good to excellent test-retest reliability across most measures (Table 1). Cervical ROM showed good to excellent reliability for all 6 directions (ICC=0.851-0.967), with low SEMs and narrow CIs, indicating stable and precise measurements. The results of JPE showed good reliability (ICC=0.813-0.827) and small MDC values (around 1.11°-1.27°), confirming the system’s ability to detect meaningful changes in repositioning accuracy. The results of FOE demonstrated good to excellent reliability, with ICCs of 0.81 for deviation frequency and 0.91 for completion duration, reflecting consistent performance across sessions. The HTR demonstrated reliability approaching the good range, with an ICC of 0.742. For PS, reliability varied by visual condition. Under eyes-open conditions, the ICC values ranged from 0.720 to 0.772, while eyes-closed conditions produced higher consistency (ICC=0.759-0.843). Overall, the VR-based system provided stable, repeatable measurements across all tests, particularly for dynamic and proprioceptive tasks.

**Table 1:** Test-retest reliability of virtual reality–based cervical sensorimotor control assessments.

		Test 1, mean (SD)	Test 2, mean (SD)	SEM^a^	MDC^b^	ICC^c^ 95% CI
**Cervical range of motion (degree)**						
	Flexion	57.65 (11.26)	57.30 (11.18)	2.07	5.73	0.966 (0.929-0.984)
	Extension	61.77 (12.96)	63.39 (13.16)	3.50	9.71	0.928 (0.850-0.966)
	Left SB^d^	40.00 (7.31)	39.78 (6.49)	1.53	4.24	0.951 (0.897-0.977)
	Right SB	44.47 (8.98)	44.9 3 (8.48)	1.56	4.33	0.968 (0.934-0.985)
	Left ROT^e^	72.93 (7.88)	74.53 (7.59)	2.81	7.79	0.868 (0.723-0.937)
	Right ROT	76.71 (7.72)	77.17 (6.58)	2.77	7.67	0.851 (0.687-0.929)
**Joint position error (degree)**						
	Extension	2.93 (1.00)	2.88 (0.90)	0.41	1.13	0.816 (0.611-0.912)
	Flexion	2.66 (0.98)	2.57 (0.98)	0.41	1.13	0.827 (0.637-0.917)
	Left ROT	3.18 (0.96)	3.28 (0.92)	0.40	1.11	0.820 (0.624-0.914)
	Right ROT	3.03 (0.92)	3.31 (1.19)	0.46	1.27	0.813 (0.608-0.911)
**Figure of eight**						
	Deviation (times)	21.34 (4.86)	20.58 (4.72)	2.09	5.79	0.810 (0.609-0.908)
	Duration (seconds)	32.82 (12.36)	30.17 (10.40)	3.37	9.34	0.913 (0.803-0.960)
Head-tilt response (degree)		3.81 (1.63)	3.79 (1.41)	0.77	2.15	0.742 (0.293-0.782)
**Postural sway: eyes-open condition**						
	AP^f^ (cm)	4.93 (2.23)	5.01 (2.20)	1.06	2.93	0.772 (0.502-0.895)
	ML^g^ (cm)	2.24 (1.19)	2.05 (1.17)	0.59	1.64	0.749 (0.484-0.879)
	TPL^h^ (cm)	41.98 (13.17)	41.32 (15.68)	7.51	20.82	0.731 (0.414-0.876)
	SA^i^ (cm^2^)	9.07 (7.90)	8.85 (10.53)	4.93	13.65	0.720 (0.387-0.871)
**Postural sway: eyes-closed condition**						
	AP (cm)	6.40 (2.54)	6.26 (2.36)	1.13	3.14	0.787 (0.536-0.902)
	ML (cm)	2.87 (1.39)	2.84 (1.42)	0.69	1.91	0.759 (0.475-0.889)
	TPL (cm)	51.27 (19.93)	51.66 (22.61)	8.44	23.41	0.843 (0.658-0.927)
	SA (cm^2^)	13.06 (12.34)	13.07 (12.74)	5.54	15.35	0.805 (0.575-0.910)

^a^SEM: standard error of measurement.

^b^MDC: minimal detectable change.

^c^ICC: intraclass correlation coefficient.

^d^SB: side bending.

^e^ROT: rotation.

^f^AP: anteroposterior direction displacement.

^g^ML: mediolateral direction displacement.

^h^TPL: total path length.

^i^SA: sway area.

Bland-Altman plots were generated for each sensorimotor domain to visualize agreement between sessions. The plots revealed acceptable limits of agreement across all tests, further confirming the consistency of the VR-based assessments (Multimedia Appendix 1).

### Concurrent Validity

Table 2 summarizes the concurrent validity results comparing VR-based cervical SMC assessments with reference measures from the motion analysis system or established clinical tests. Overall, VR outcomes showed strong to excellent correlations with their gold-standard counterparts, confirming concurrent validity across the domains for ROM, JPE, FOE, and PS. For cervical ROM, excellent correlations were observed between VR and Vicon data across 6 movement directions (*r*=0.892-0.980), indicating highly comparable ROM outputs. The results of JPE demonstrated strong to excellent correlations with Vicon-based angular deviations (*r*=0.869-0.953), confirming accurate detection of proprioceptive errors. The FOE test demonstrated an excellent correlation for deviation frequency (*r*=0.923) and a strong correlation for task completion duration (*r*=0.723) compared with corresponding clinical reference measures, supporting its validity in assessing dynamic head coordination. For PS, VR-derived sway metrics showed strong correlations with Vicon-based measures under both visual conditions, with the highest agreement observed for TPL (*r*=0.895-0.917) and SA (*r*=0.908-0.962). These findings collectively demonstrate that the VR-based system provides accurate and valid measures of cervical motion, proprioception, coordination, and postural stability.

**Table 2:** Concurrent validity of virtual reality (VR)-based cervical sensorimotor control assessments.

		VR, mean (SD)	Standard reference, mean (SD)	*r*
**Cervical range of motion (degree)**				
	Flexion	49.82 (12.97)	54.77 (13.20)	0.892
	Extension	58.89 (12.57)	53.82 (12.53)	0.917
	Left SB^a^	39.31 (6.85)	39.14 (7.40)	0.958
	Right SB	42.85 (9.21)	42.31 (7.61)	0.927
	Left ROT^b^	72.96 (8.53)	71.50 (8.50)	0.980
	Right ROT	75.33 (7.27)	76.12 (7.54)	0.946
**Joint position error (degree)**				
	Extension	4.19 (1.53)	3.52 (1.55)	0.882
	Flexion	4.28 (2.72)	3.56 (2.66)	0.953
	Left ROT	3.83 (1.91)	3.32 (1.61)	0.869
	Right ROT	3.50 (1.24)	2.79 (1.23)	0.920
**Figure of eight**				
	Deviations (times)	21.83 (4.63)	32.43 (7.44)	0.923
	Durations (seconds)	30.94 (8.27)	31.13 (8.67)	0.723
**Postural sway: eyes-open condition**				
	AP^c^ (cm)	5.03 (2.41)	2.41 (1.01)	0.801
	ML^d^ (cm)	2.56 (1.25)	1.04 (0.47)	0.871
	TPL^e^ (cm)	41.45 (14.41)	14.06 (5.03)	0.895
	SA^f^ (cm^2^)	9.48 (9.49)	1.82 (1.79)	0.962
**Postural sway: eyes-closed condition**				
	AP (cm)	6.70 (3.22)	3.84 (2.15)	0.825
	ML (cm)	2.69 (1.27)	1.21 (0.59)	0.851
	TPL (cm)	49.64 (20.49)	20.67 (10.25)	0.917
	SA (cm^2^)	12.81 (13.40)	3.54 (5.11)	0.908

^a^SB: side bending.

^b^ROT: rotation.

^c^AP: anteroposterior direction displacement.

^d^ML: mediolateral direction displacement.

^e^TPL: total path length.

^f^SA: sway area.

## Discussion

### Principal Findings

This study demonstrated that a custom-developed VR-based system provided reliable assessments of cervical SMC across all domains (ROM, JPE, FOE, HTR, and PS) and valid measurements for ROM, JPE, FOE, HTR, and PS. The shows that this VR-based system exhibited good to excellent test-retest reliability. The consistency of repeated measurements was further supported by Bland-Altman plot analyses. Concurrent validity revealed strong correlations with gold-standard measures for ROM, JPE, FOE, and PS, confirming the measurement accuracy of this system in assessing cervical SMC. These results are consistent with previous studies supporting the reliability and validity of VR-based approaches for assessing cervical ROM and proprioception [24,46-50]. Prior work has demonstrated strong agreement between VR-derived and optical or goniometric measurements, as well as good to excellent reliability using IMU-based or VR-based systems [24,46-52]. A recent systematic review also confirmed that VR-based ROM assessments effectively differentiate between individuals with and without CNP [24,46,49]. In this study, the neutral head position was standardized using a calibration procedure; however, slight variations in self-selected posture may still influence absolute ROM values. Repeated calibration before each trial and consistent procedures across sessions likely minimized systematic bias and contributed to the high test-retest reliability observed.

Compared with previous VR-based cervical assessment systems, this study offers several methodological and conceptual advancements. Most previous studies have focused on single-domain assessments [53], whereas the current system integrates multiple domains of cervical SMC, including mobility, proprioception, coordination, verticality perception, and postural stability, within a single immersive platform. In addition, several tasks were specifically designed for VR-based interaction and automated data capture, enabling continuous kinematic tracking without reliance on manual scoring. Furthermore, this study systematically evaluated both test-retest reliability and concurrent validity across all domains within the same cohort, providing a more comprehensive validation framework.

This study also presents an immersive VR adaptation of the FOE head-tracing task, enabling quantitative assessment of coordination through metrics, such as completion time and deviation frequency. Compared with traditional head-mounted laser-pointer methods [36,47], the VR implementation allows standardized visual presentation, continuous trajectory tracking, and automated outcome computation, thereby reducing examiner dependency and improving measurement precision. Furthermore, the VR-based format allows controlled manipulation of visual feedback and task complexity, offering new opportunities to quantify SMC across varying sensory conditions. Similarly, the HTR task demonstrated acceptable reliability, consistent with previous studies on VR-based assessments of verticality perception [7,14,25,54-56].

The VR-based PS assessment exhibited good reliability and strong correlations with reference measures under both visual conditions, consistent with previous findings [57,58]. Previous studies have also reported moderate to strong agreement between VR-based and force plate-based PS measures, supporting the use of VR systems as portable and cost-effective tools for clinical balance assessment [59]. In addition, VR-based sway metrics have been shown to differentiate between healthy individuals and clinical populations across various applications [60-62]. However, systematic differences in absolute magnitude were observed, with VR values consistently higher than those obtained from the reference system. These discrepancies may be attributed to fundamental differences in measurement principles, as the VR-based sway metrics were reconstructed from head-mounted IMU signals, whereas the reference system directly quantified head displacement using marker-based motion capture. In addition, IMU-based approaches are inherently more sensitive to high-frequency motion components and may accumulate small fluctuations in orientation signals, leading to overestimation of trajectory-based metrics such as TPL and SA. Similar patterns have been reported in previous studies, where strong correlations were observed despite differences in absolute values [39,40]. These findings suggest that a VR-based system is suitable for relative comparisons and longitudinal monitoring, although direct interchangeability with reference systems should be interpreted with caution.

Slightly lower reliability observed in the HTR and certain PS parameters is consistent with previous studies and likely reflects the intrinsic variability of perceptual, proprioceptive, and balance-related tasks rather than limitations of the VR system itself [47,49]. Such variability, influenced by sensory integration, attentional demand, and postural control strategies across sessions, may reduce test-retest stability even under standardized conditions. Nevertheless, the observed reliability levels remain within acceptable ranges for complex sensorimotor assessments.

From a clinical measurement perspective, the relatively small SEM and MDC values observed across most parameters indicate good measurement precision and support the ability to detect meaningful changes at the individual level. MDC values provide a practical threshold for distinguishing true change from measurement error, suggesting that relatively small improvements can be confidently interpreted as real changes rather than measurement variability. These findings support the use of the VR-based system for monitoring rehabilitation progress and evaluating intervention outcomes. Taken together, the present findings support the notion that the proposed VR system provides an integrated and clinically feasible platform for the multidimensional assessment of cervical SMC. Furthermore, the high repeatability observed in healthy participants may also serve as a normative reference for cervical SMC performance. Such baseline data could facilitate future comparisons with individuals presenting with cervical symptoms or pathologies, thereby supporting the clinical applicability of the VR-based system for assessment and rehabilitation.

### Limitations

Despite the valuable contributions of this study, several limitations should be acknowledged. The participants in this study were healthy young adults; therefore, the results may not generalize to older populations or those with CNP. Future research should validate the system in diverse age groups and clinical populations, and further examine its predictive validity and responsiveness to intervention-induced changes. Although participants were instructed to minimize trunk movement and were visually monitored during testing, compensatory cervicothoracic or trunk motion cannot be completely excluded, particularly during lateral bending tasks. As the current system relies on head-mounted tracking without additional trunk sensors, the measured angles may partially reflect combined head and trunk motion. Future studies incorporating objective trunk motion monitoring (eg, thoracic IMUs, motion capture markers, or head-to-trunk relative angle calculations) are warranted to further isolate cervical motion and improve measurement specificity. Despite this limitation, the high test-retest reliability observed in this study suggests that the measurement approach was consistent across sessions. Visual requirements may pose a practical constraint during the VR-based assessment. Participants who usually wore glasses were required to use contact lenses to ensure compatibility with the VR headset, which may have excluded individuals with visual impairments or low tolerance for immersive VR environments. This limitation may introduce potential bias and affect both the accessibility and ecological validity of the system in real-world clinical applications. Although familiarization trials were provided prior to formal testing, the novelty of the VR environment may have introduced learning effects or interindividual variability in task adaptation, potentially influencing performance consistency across trials and participants. Moreover, factors such as headset fit, visual delay, or cybersickness could affect measurements, though none were reported in this study. Continued optimization of head-tracking accuracy and algorithm calibration may further enhance the system’s sensitivity to low-amplitude motion and subtle postural adjustments.

### Conclusion

This study established the reliability and validity of a novel VR-based assessment system for the multidimensional assessment of cervical SMC. A single session provides data across 4 major SMC domains and the immersive environment sustains participant motivation. The system demonstrated good to excellent reliability across all domains and strong concurrent validity for ROM, JPE, FOE coordination, and PS. Its standardized, immersive, and user-friendly design supports efficient and objective evaluation of cervical sensorimotor function with reduced operator bias and higher reproducibility. Future studies should extend validation to clinical populations and evaluate responsiveness to therapeutic interventions. This VR-based platform uses commercially available hardware and portable software that is adaptable for both clinical and tele-assessment contexts and provides a robust foundation for establishing normative reference values and advancing digital assessment and management of CNP in both clinical and telehealth settings.

## Data Availability

The datasets generated or analyzed during this study are available from the corresponding author on reasonable request.

## Appendix

Multimedia Appendix 1 Bland-Altman plots for agreement between repeated virtual reality–based assessments.

## Abbreviations

CNP: chronic neck pain
FOE: figure of eight
HTR: head-tilt response
ICC: intraclass correlation coefficient
IMU: inertial measurement unit
JPE: joint position error
MDC: minimal detectable change
PS: postural sway
ROM: range of motion
SA: sway area
SEM: standard error of measurement
SMC: sensorimotor control
TPL: total path length
VR: virtual reality
