# Supplementary material for: Validity and Reliability of an Immersive Virtual Reality System for Multidimensional Assessment of Cervical Sensorimotor Control: Cross-Sectional Study
Source: JMIR Rehabil Assist Technol. 2026 May 21;13:e88498. doi: 10.2196/88498 (PMC13237531; doi:10.2196/88498)
Supplement: Multimedia Appendix 1 [file rehab_v13i1e88498_app1.pdf]

Bland-Altman Plots for Agreement between Repeated VR-based Assessment

This appendix presents Bland–Altman plots illustrating the agreement between repeated sessions for each VR-based assessment domain. Each plot displays the mean difference (bias) and 95% limits of agreement (mean ± 1.96 × SD of the differences) between test and retest measurements. The results demonstrate minimal systematic bias and acceptable limits of agreement across the cervical range of motion (CROM), joint position error (JPE), figure-of-eight (FOE), head-tilt response (HTR), and postural sway (PS) assessments, supporting the stability and reliability of the VR-based system.

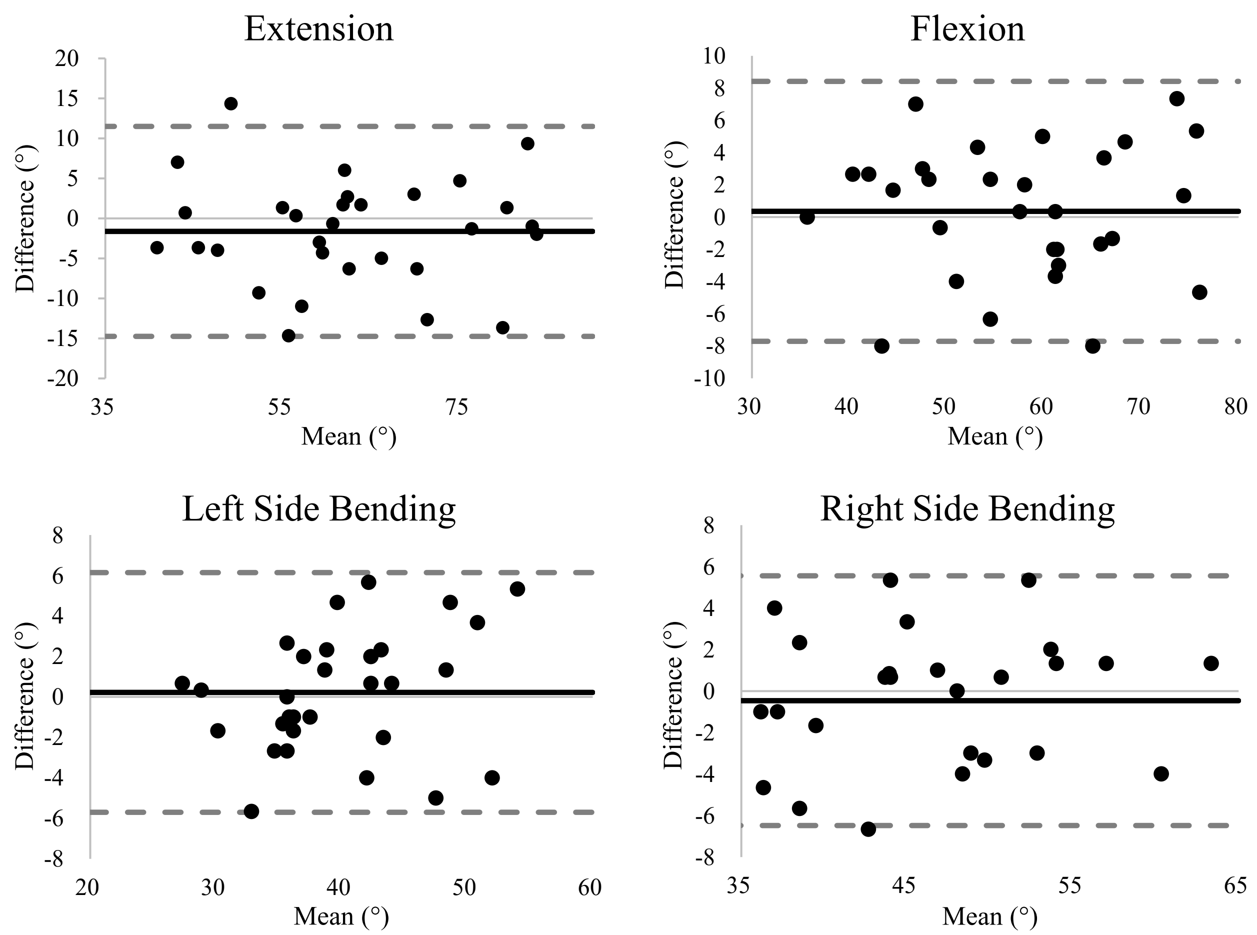

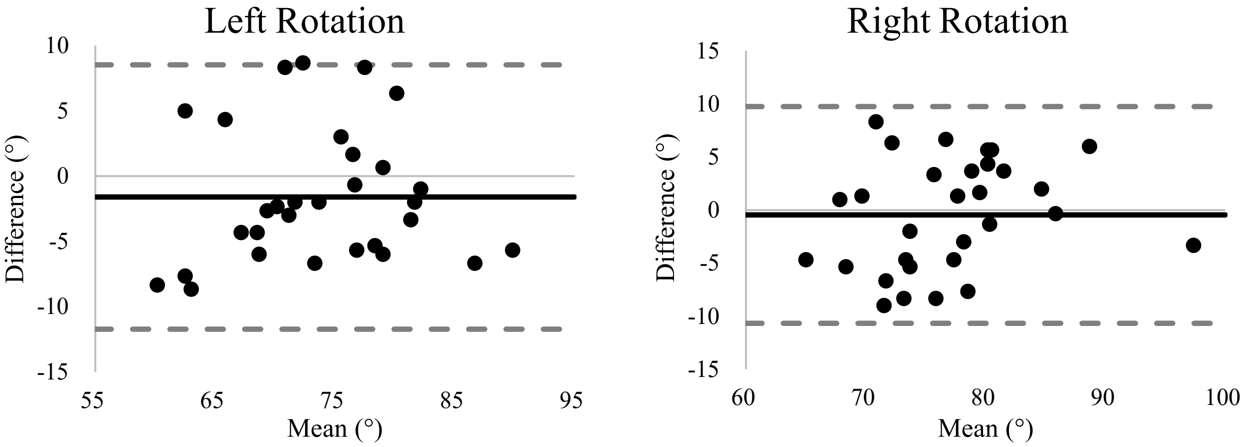

(A) Bland-Altman Plots for Cervical Range of Motion

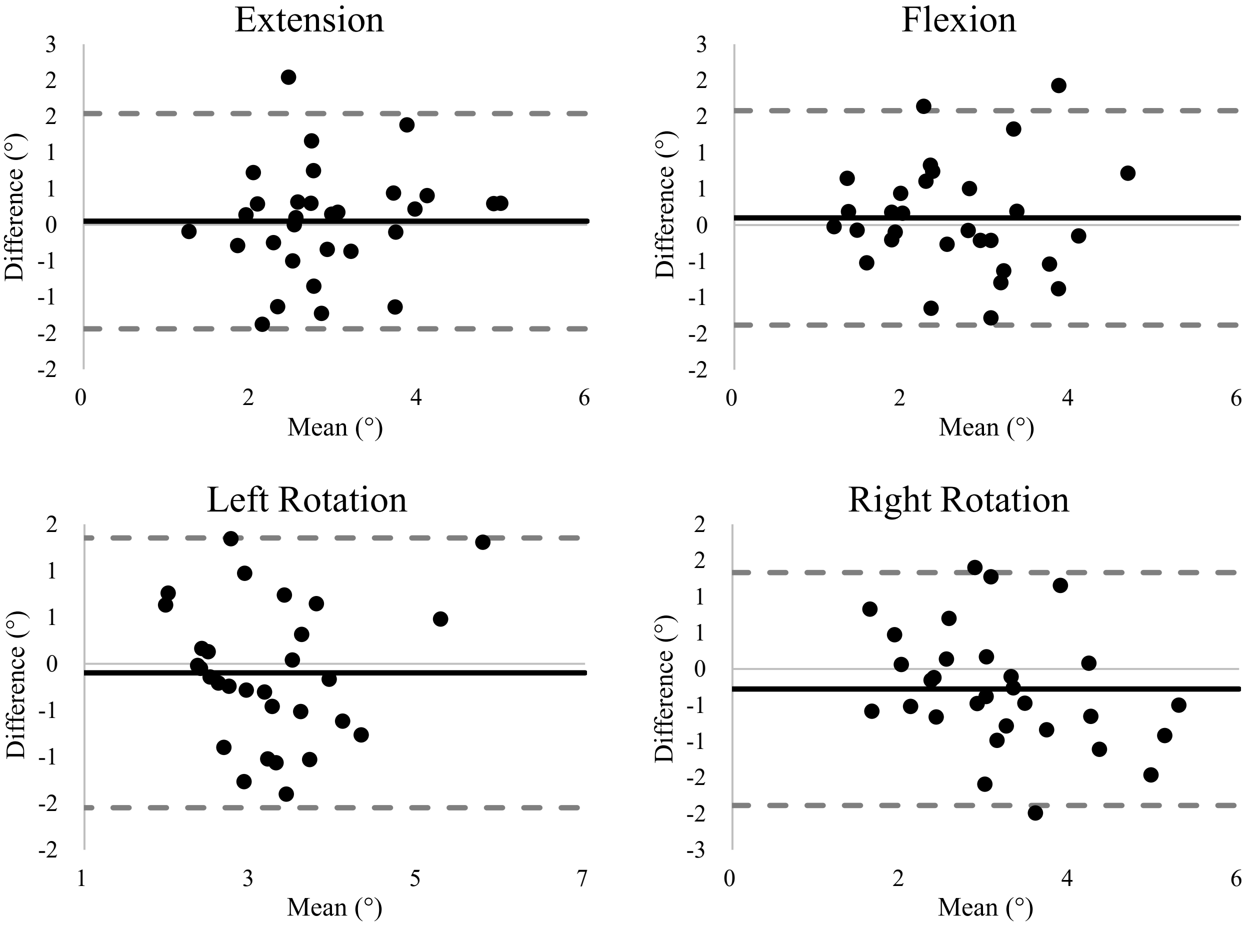

(B) Bland-Altman Plots for Joint Position Error

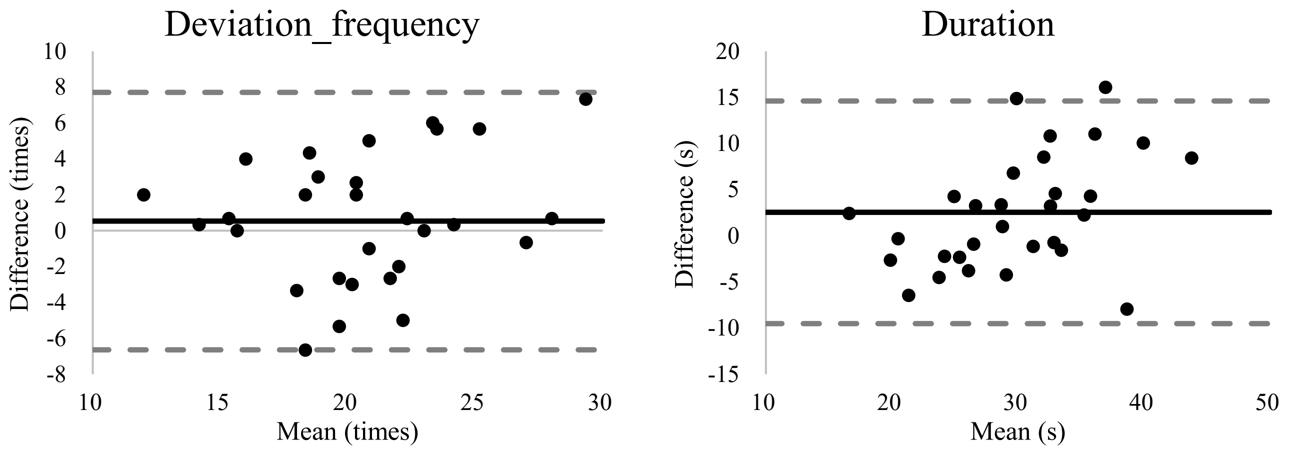

(C) Bland-Altman Plots for Figure of Eight

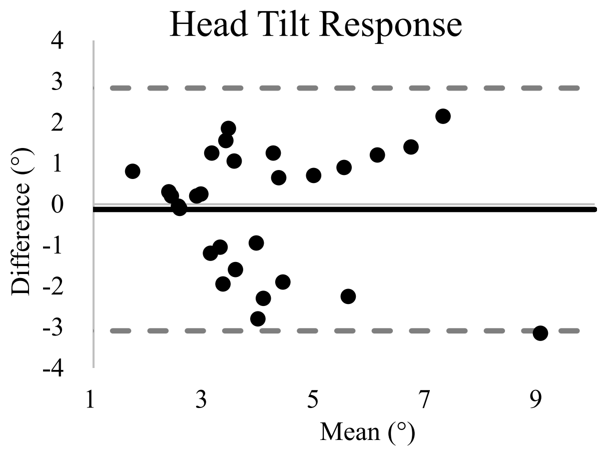

(D) Bland-Altman Plots for Head Tilt Response

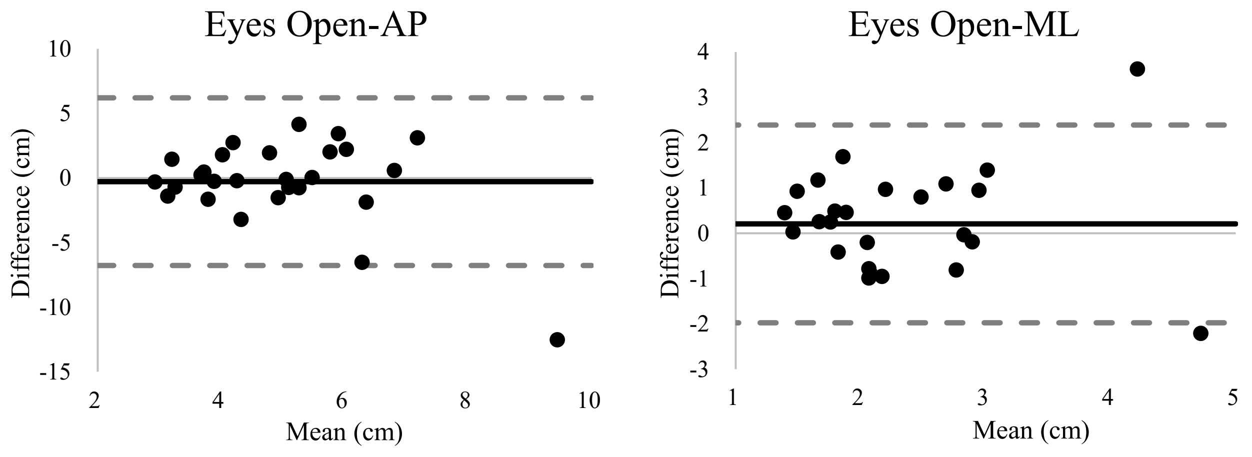

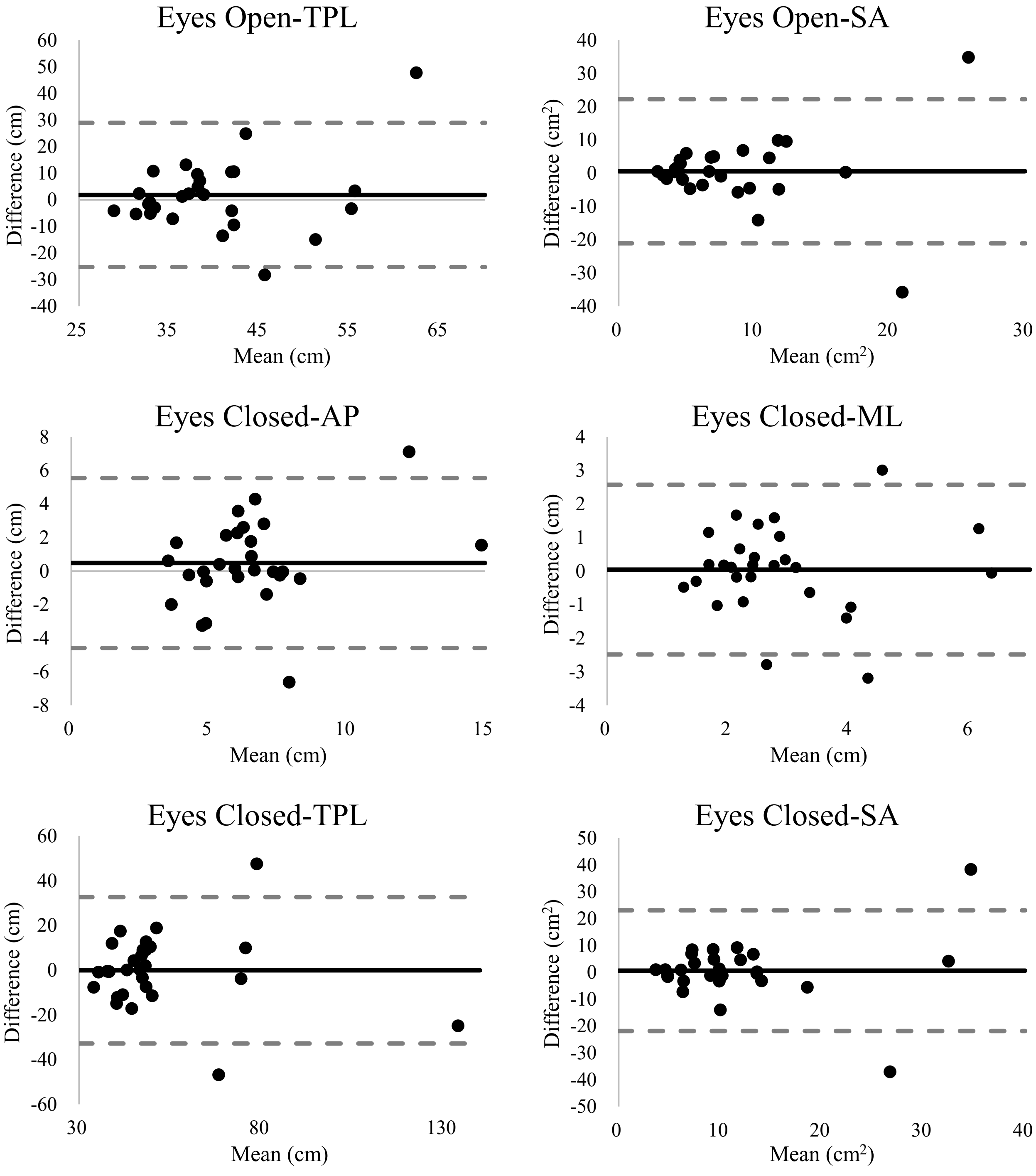

(E) Bland-Altman Plots for Postural Sway in Eyes Open & Closed Conditions
